# Thymosin β4 Identified by Transcriptomic Analysis from HF Anagen to Telogen Promotes Proliferation of SHF-DPCs in Albas Cashmere Goat

**DOI:** 10.3390/ijms21072268

**Published:** 2020-03-25

**Authors:** Bai Dai, Fei Hao, Teng Xu, Bing Zhu, Li-Qing Ren, Xiao-Yu Han, Dong-Jun Liu

**Affiliations:** State Key Laboratory of Reproductive Regulation and Breeding of Grassland Livestock, School of Life Sciences, Inner Mongolia University, Hohhot 010070, China; daibai@mail.imu.edu.cn (B.D.); feihao@imu.edu.cn (F.H.); xuteng@wmu.edu.cn (T.X.); nmzhubing@mail.imu.edu.cn (B.Z.); renliqing@mail.imu.edu.cn (L.-Q.R.); hanxiaoyu@mail.imu.edu.cn (X.-Y.H.)

**Keywords:** thymosin beta-4 (Tβ4), cashmere goat, hair follicle (HF), SHF-DPCs, proliferation

## Abstract

Increasing cashmere yield is one of the important goals of cashmere goat breeding. To achieve this goal, we screened the key genes that can improve cashmere performance. In this study, we used the RNA raw datasets of the skin and dermal papilla cells of secondary hair follicle (SHF-DPCs) samples of hair follicle (HF) anagen and telogen of Albas cashmere goats and identified a set of significant differentially expressed genes (DEGs). To explore potential associations between gene sets and SHF growth features and to identify candidate genes, we detected functional enrichment and constructed protein-protein interaction (PPI) networks. Through comprehensive analysis, we selected *Thymosin β4* (*Tβ4*), *Rho GTPase activating protein 6* (*ARHGAP6*), *ADAM metallopeptidase with thrombospondin type 1 motif 15*, (*ADAMTS15*), *Chordin* (*CHRD*), and *SPARC (Osteonectin), cwcv* and *kazal-like domains proteoglycan 1* (*SPOCK1*) as candidate genes. Gene set enrichment analysis (GSEA) for these genes revealed *Tβ4* and *ARHGAP6* have a close association with the growth and development of SHF-DPCs. However, the expression of Tβ4 in the anagen was higher than that in the telogen, so we finally chose Tβ4 as the ultimate research object. Overexpressing Tβ4 promoted and silencing Tβ4 inhibited the proliferation of SHF-DPCs. These findings suggest that Tβ4 can promote the growth and development of SHF-DPCs and indicate that this molecule may be a valuable target for increasing cashmere production.

## 1. Introduction

Cashmere is an important economic product worldwide and the world market for cashmere is increasing, but the current production of cashmere is limited [1,2,3,4]. Albas cashmere goat, famous across the world for its high-quality cashmere, is one of the most precious genetic resources on the Inner Mongolia Plateau in China [5,6,7]. However, due to the failure to form a scientific germplasm conservation awareness for this species, the population resources show a trend of degradation. Therefore, the use of modern biological breeding technology to breed high-quality and high-yield cashmere goats has become an effective method to protect and use the germplasm resources. Cashmere goats harbor two different kinds of fleece: A short and fine nonmedullated cashmere fiber, which is produced by a secondary hair follicle (SHF), and a long and coarse medullated guard hair, which is produced by a primary hair follicle (PHF) [8]. Hair follicle (HF) growth, which is a highly orchestrated and cyclic process, involves three main stages: Anagen (the rapid growth phase, from April until November), catagen (the gradual degeneration phase, from December to January), and telogen (the relative static phase, from February to March) [9,10,11,12,13]. Although the hair follicle cycle stage of cashmere goat has a unique length and duration, the basic principle of the cyclic transformations of hair follicles is similar to other mammals. The cross talk between different types of cells in the hair follicle during proliferation, differentiation, and apoptosis is the cellular basis of periodic HF regeneration [14,15].

Dermal papilla cells (DPCs), the specialized mesenchymal components of HF, play an important role in the morphogenesis and regeneration of HF. As a reservoir of pluripotent stem cells, nutrients, and growth factors, DPCs regulate the development and growth of HF and effect on the HF cycle [16]. DPCs promote the differentiation of hair follicle stem cells (HFSCs) into HF during the periodic transition. At the telogen stage, HFSCs are located in the bulge of the HF. In the process of HF transformation from telogen to anagen, DPCs migrate upward and then reach the bulge. In the bulge, DPCs release signals to activate multiple signaling pathways, especially the Wnt/β-catenin signaling pathway [17], stimulate the differentiation of HFSCs, and promote HF regeneration [18]. In cashmere goat, dermal papilla cells of secondary hair follicle (SHF-DPCs) are the controlling centers of SHF growth. In recent years, SHF-DPCs have been the focus of intense interest for cashmere goat researchers. Due to their important role in cashmere growth, SHF-DPCs serve as an excellent model to investigate the function mechanism of genes on HF in cashmere goats [19,20,21,22]. Therefore, to improve the individual cashmere production of cashmere goats, researchers tried to find the major genes and pathways that may affect the growth and development of SHF-DPCs and HF cycle by transcriptomics and bioinformatics methods [4,12,23].

Thymosin beta-4 (Tβ4), a 4.9 kDa actin sequestering protein, was originally isolated from thymic extract [24]. The study of Tβ4 has gradually become a research hotspot, mainly due to its involvement in a variety of biological processes. In 1995, Grant et al. reported that Tβ4 is involved in endothelial cell differentiation [25]. In addition to its ability to induce endothelial cell differentiation, Tβ4 promotes endothelial cell migration and stimulates microtubule formation and angiogenesis in vitro and in vivo. Tβ4 enhances wound healing through various mechanisms, including increased angiogenesis, improved keratinocyte migration, collagen deposition, and wound contracture [26,27]. Tβ4 has anti-inflammatory and hair growth improvement effects [27,28,29]. Tβ4 has been shown to play key roles in HF growth and development [30]. Endogenous Tβ4 can activate the HF cycle transition in mice, and affects HF growth and development by promoting the migration and differentiation of HFSCs and their progeny [31,32,33,34]. In addition, exogenous Tβ4 increases the rate of hair growth in mice and promotes cashmere production by increasing the number of SHF in cashmere goats [35,36]. Taken together, these findings demonstrate that Tβ4 is a pleiotropic protein that plays important roles in the growth and development of HF by affecting the growth and development of HF through its various functions.

In this study, we analyzed RNA-sequencing (RNA-seq) raw data and found that Tβ4 plays an important role in SHF of Albas cashmere goats by comparing the SHF-DPCs samples with the skin samples from anagen to telogen. We used SHF-DPCs as an in vitro model of SHF and investigated the effects of Tβ4 on the proliferation of SHF-DPCs, and then determined the function of Tβ4 in the regulation of HF growth and development of cashmere goats, which shed light on further research for the mechanism of Tβ4 in this process.

## 2. Results

### 2.1. Selection of Genes from RNA-Seq from HF Anagen to Telogen of Albas Cashmere Goat

**Figure 1:**
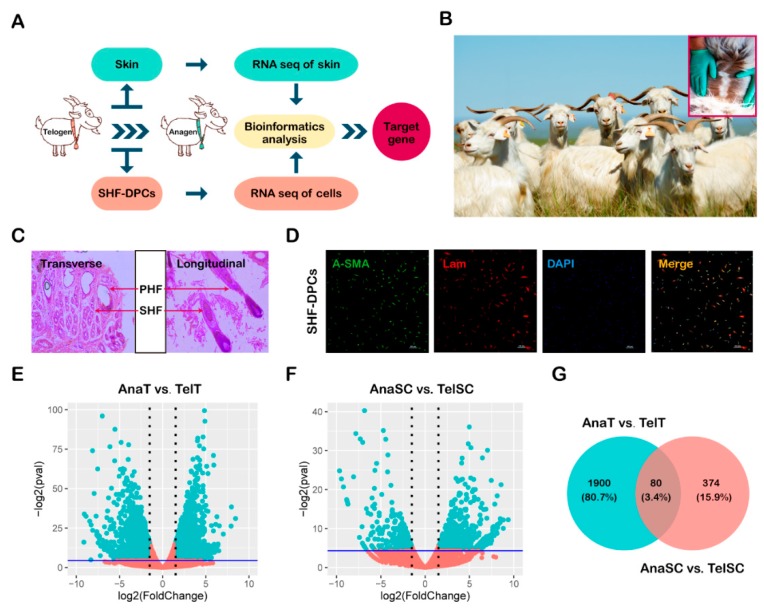
Transcriptomic analysis of overlapping differentially expressed genes (DEGs) from hair follicle (HF) anagen to telogen of Albas cashmere goat. (**A**) Our experimental workflow. (**B**) Albas cashmere goats living on the Inner Mongolia Plateau. The red frame shows where cashmere grows. (**C**) Hematoxylin-eosin (H&E) staining of skin in transverse and longitudinal section. Arrows indicate the primary hair follicle (PHF) and the secondary hair follicle (SHF). (**D**) Identification of dermal papilla cells of secondary hair follicle (SHF-DPCs) using anti-α smooth muscle actin (α-SMA) (green) antibody and anti-laminin antibody (red). SHF-DPCs were positive for both antibodies and nuclei were marked by 4′,6-diamidino-2-phenylindole (DAPI) staining (blue). (**E–F**) Volcano plot of all genes in the skin and SHF-DPCs samples of HF anagen and telogen of Albas cashmere goat, showing genes with >2-fold difference and an adjusted *p* < 0.01 among groups. AnaT, the skin tissue of HF anagen; TelT, the skin tissue of HF telogen, AnaSC, the SHF-DPCs of HF anagen, TelSC, the SHF-DPCs of HF telogen. (**G**) Venn diagram illustrating the number of overlapping differentially expressed genes (overlapping DEGs) between AnaT vs. TelT and AnaSC vs. TelSC.

Figure 1A shows our workflow for gene selection. To identify the potential genes affecting the HF growth cycle of Albas cashmere goat, we first quantified the whole genome transcriptomes of skin tissue (Figure 1B,C) and SHF-DPCs (Figure 1D) at the catagen and telogen stages of HF in three female Albas cashmere goats. We defined 1980 differentially expressed genes (DEGs) between the skin tissue at the telogen and catagen stages, consisting of 1119 downregulated genes and 861 upregulated genes (Figure 1E). We defined 454 DEGs between SHF-DPCs at the telogen and catagen stages, consisting of 166 downregulated and 288 upregulated genes (Figure 1F). The overlapping DEGs among the two datasets contained 80 genes, as shown in the Venn diagram in Figure 1G. Further analysis of overlapping DGEs revealed 66 genes encoding proteins, 4 genes encoding RNA, 2 genes encoding pseudogenes, and 5 genes encoding uncharacterized proteins. Therefore, 66 protein-coding genes were selected for further screening.

### 2.2. Functional Enrichment and Protein-Protein Interaction (PPI) Construction of Selected DEGs

**Figure 2:**
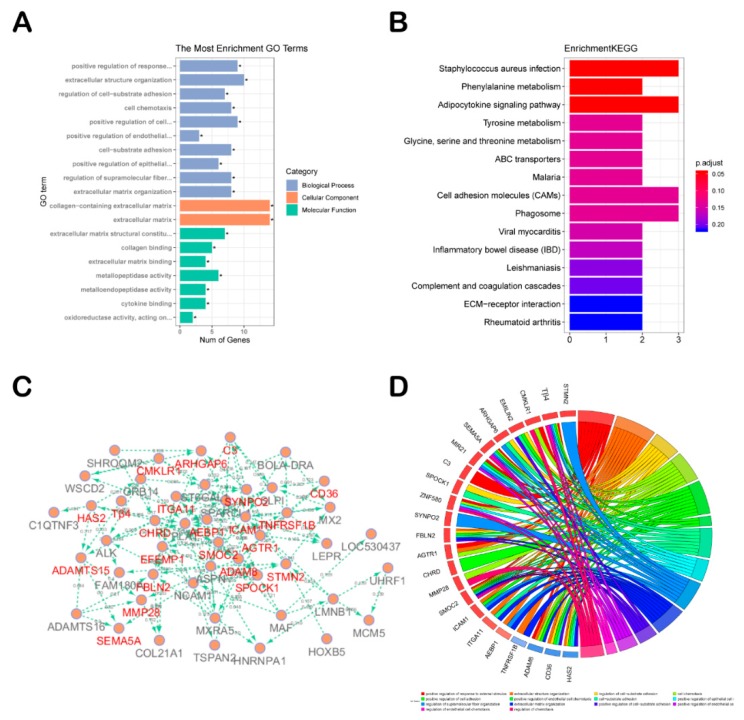
Functional characterization and protein-protein interaction (PPI) analysis of selected DEGs from HF anagen to telogen of Albas cashmere goat. (**A**) Gene ontology (GO) and (**B**) encyclopedia of genes and genomes (KEGG) analyses of selected DEGs. (**C**) PPI network analysis of selected DEGs. The genes in red are those in the Chord plot in Figure 2D. (**D**) Chord plot depicting the relationship between genes and GO terms of biological process.

The 66 protein-coding genes, as selected DEGs, were chosen to perform gene ontology (GO) and encyclopedia of genes and genomes (KEGG) analyses. With GO analysis, we detected enrichment in several biological process GO terms, such as positive regulation of response to external stimulus, extracellular structure organization, regulation of cell substrate adhesion, cell chemotaxis, and positive regulation of cell adhesion. In terms of cellular component, collagen-containing extracellular matrix was the most significantly enriched GO term. In some molecular component GO terms, such as collagen binding and extracellular matrix, structural constituent was enriched (Figure 2A). For KEGG pathway analysis, *staphylococcus aureus* infection, phenylalanine metabolism, and adipocytokine-signaling pathway were mostly associated with these genes (Figure 2B). The PPI network of these genes is shown in Figure 2C and most hub genes were consistent with the genes involved in biological processes GO term (Figure 2D). Given the above comprehensive analysis, we chose *Thymosin β4* (*Tβ4*), *Rho GTPase activating protein 6* (*ARHGAP6*), *ADAM metallopeptidase with thrombospondin type 1 motif 15*, (*ADAMTS15*), *Chordin* (*CHRD*), and *SPARC (Osteonectin), cwcv and kazal-like domains proteoglycan 1* (*SPOCK1*) as candidate genes.

### 2.3. Analysis of Relationship between Candidate Genes and Hair Follicle Growth

**Figure 3:**
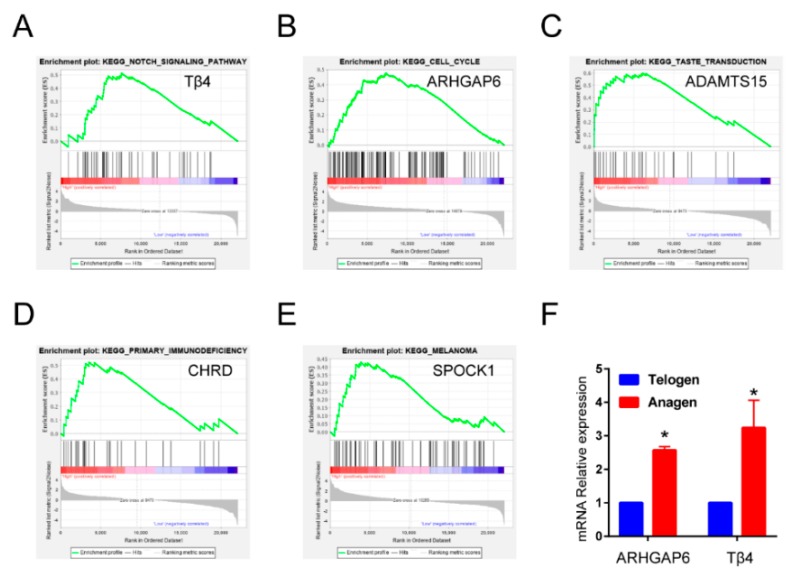
Screening of candidate genes from HF anagen to telogen stages of Albas cashmere goat. (**A–E**) Gene set enrichment analysis (GSEA) analysis of five candidate genes. (**F**) The two candidate genes most relevant to hair follicle growth were identified by quantitative real-time PCR (qPCR).

The abbreviations, full names, and descriptions for candidate genes are provided in Table 1. To further investigate the potential functions of *Tβ4*, *ARHGAP6*, *ADAMTS15*, *CHRD*, and *SPOCK1* in HF of cashmere goat, we performed gene set enrichment analysis (GSEA) on the skin tissue RNA-seq data. As shown in Figure 3A–E, in the pathways associated with HF, genes in high-expression groups of *Tβ4* were enriched in notch signaling pathways. The cell cycle and taste transduction gene sets were enriched in high-expression groups of *ARHGAP6* and *ADAMTS15,* respectively, whereas primary immunodeficiency and melanoma were enriched in the *CHRD* and *SPOCK1* high-expression groups, respectively. Then, we chose Tβ4 and ARHGAP6 as targets for further identification. Because gene sets with the highest enrichment scores were more closely associated with HF growth in high-expression groups of *Tβ4* and *ARHGAP6*, we further validated the RNA-seq results of *Tβ4* and *ARHGAP6*. Their expression patterns in anagen and telogen stages of the HF were examined using quantitative real-time PCR (qPCR). Expression patterns were consistent with expression levels calculated from the RNA-seq data (Figure 3F). Compared with *ARHGAP6*, the expression of *Tβ4* in the anagen stage was higher than in the telogen stage, so we finally chose Tβ4 as the ultimate research object.

### 2.4. Identification of Tβ4 Promoting Proliferation of SHF-DPCs

**Figure 4:**
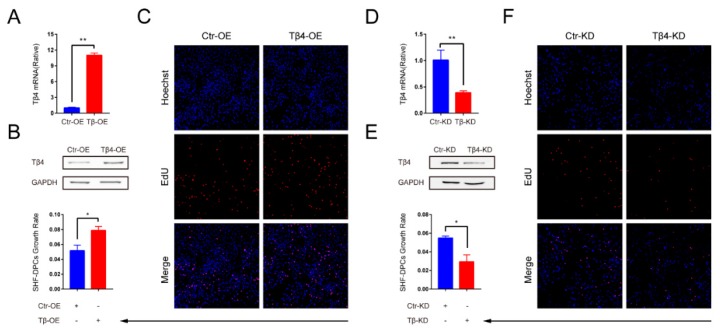
The growth rate of SHF-DPCs was investigated by transfecting Tβ4 overexpression (Tβ4-OE) or Tβ4 knockdown (Tβ4-KD) vector. (**A**) qPCR and (**B**) Western blot were used to verify the overexpression efficiency of Tβ4. SPH-DPCs transfected control for Tβ4 overexpression (Ctr-OE) and Tβ4-OE vector (** *p* < 0.01). (**C**) The 5-ethynyl-2′-deoxyuridine (EdU) assays revealed that overexpression of Tβ4 significantly increased the growth rate of SPH-DPCs (* *p* < 0.05). (**D**) qPCR and (**E**) Western blot were used to verify the interference efficiency of Tβ4. SPH-DPCs transfected control for Tβ4 knockdown (Ctr-KD) and Tβ4-KD vector (** *p* < 0.01). (**F**) EdU assays revealed that downregulation of Tβ4 significantly reduced the growth rate of SPH-DPCs (* *p* < 0.05) (n.s, not significant).

**Figure 5:**
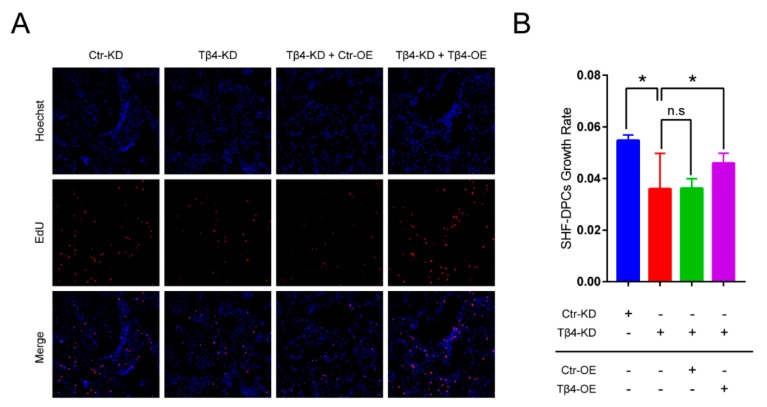
The growth rate of Tβ4-KD-SHF-DPCs was rescued by transfecting Tβ4-OE vector. (**A**) EdU staining was used to assess the proliferation of SHF-DPCs. (**B**) The percentage of EdU-positive SHF-DPCs was calculated (* *p* < 0.05).

To verify whether Tβ4 can promote hair follicle growth, we ectopically overexpressed Tβ4 in SHF-DPCs (Figure 4A,B).The 5-ethynyl-2′-deoxyuridine (EdU) assays revealed that overexpression of Tβ4 (Tβ4-OE) significantly increased the SHF-DPCs numbers, which were approximately 1.6-fold higher after plating compared with vector control cells (Figure 4C). We inhibited Tβ4 in SHF-DPCs (Figure 4D,E). EdU assays revealed that knockdown of Tβ4 (Tβ4-KD) significantly reduced the proliferation of SHF-DPCs (Figure 4F). We also ectopically overexpressed Tβ4 in Tβ4-KD-SHF-DPCs. EdU assays revealed that overexpression of Tβ4 significantly increased the Tβ4-KD-SHF-DPCs numbers, which were reduced by knockdown of Tβ4 (Figure 5). These results indicated that Tβ4 can enhance the proliferation of SHF-DPCs and may promote HF growth of cashmere goats.

## 3. Discussion

Increasing cashmere yield is one of the important goals of cashmere goat breeding. Screening the key genes that can improve cashmere performance is an important method for achieving this goal. We used RNA-seq raw datasets of the skin and SHF-DPCs samples of SHF anagen and telogen stages of Albas cashmere goat to comprehensively screen for key genes. Our three main reasons for choosing this analysis strategy were, first, because the HF of cashmere goat has annual periodicity. The anagen stage of HF occurs from April until November, the catagen stage is from December to January, and the telogen stage from February to March [37]. Among them, the anagen and telogen stages are the most different in the growth and development of SHF. This phenomenon must be regulated by multiple genes [12,37]. Therefore, we thought that the DEGs between the two periods were most likely the key genes involved in the regulation of hair follicle growth and development. Second, we analyzed DEGs from skin samples and cell samples of HF anagen and telogen stages, and then further analyzed the overlap of DEGs of the two sample groups. This screening strategy not only further narrowed the screening scope [38], but, more importantly, the genes of the overlap, which form the upstream gene set involved in the regulation of the growth and development of SHF, are theoretically involved in the whole process of the growth and development of skin. Third, the RNA-seq raw data of the skin we used for our analysis were obtained prior to the publication of the goat genome sequence, which was de novo assembled [39]. The RNA-seq raw data of SHF were also analyzed using the old analysis methods [23]. Therefore, room for error existed in our previous analysis. In our study, we used the latest goat genome as the reference genome and combined it with the new analysis algorithm to reanalyze the two sample groups. Therefore, this analysis strategy guaranteed the accuracy of functional gene screening.

To the best of our knowledge, our work is the first to use the skin samples combined with SHF-DPCs samples to explore novel hub genes associated with the growth and development of SHF. Consistent with published data, the enrichment of these DEGs in several GO terms, such as extracellular structure organization, cell chemotaxis, cellular component, collagen-containing extracellular matrix, and collagen binding, confirms their involvement in the growth and development of HF [39,40,41,42]. Enrichment of the identified DEGs in some KEGG pathways, such as *Staphylococcus aureus* infection, phenylalanine metabolism, and adipocytokine-signaling pathway, also suggests their relevance in the growth and development of HF. PPI analysis showed that some genes are located at the center of the PPI network, belonging to the hub gene, such as *Tβ4*, *ARHGAP6*, *ADAMTS15*, *CHRD,* and *SPOCK1*. Based on the results of GO, KEGG, and PPI analyses, we suggest that these five genes are most closely associated with the growth and development of HF. Notably, Tβ4 is involved in multiple signaling pathways in biological processes GO term, so Tβ4 may play a key role in regulating hair follicle growth and development. To further explore these genes’ biological functions, we conducted GSEA for each candidate gene. Because gene sets with the highest enrichment scores were more closely associated with HF growth in high-expression groups of *Tβ4* and *ARHGAP6*, we further validated the RNA-seq results of *Tβ4* and *ARHGAP6*. Combined with some reports [30,36,43], we chose Tβ4 as the target gene for the study.

For hair regeneration, cellular and cell-derived components have become increasingly studied and integrated into clinical practice. According to different sources, the research on hair regeneration can be divided into three directions: Adipose-derived, blood-derived, and HF-derived. For adipose-derived research [44,45,46], the components that are typically used for aesthetic and dermal applications consist of nanofat, stromal vascular fraction cells (SVFs), adipose-derived mesenchymal stem cells (AD-MSCs), and extracellular vesicle (EVs), which have all shown capability to repair, regenerate, and rejuvenate surrounding tissue. For blood-derived research [47,48,49,50,51], studies focused on platelet-rich plasma (PRP), which is a whole-blood centrifuged concentrate that contains proteins and growth factors (GFs) but not red blood cells. However, although many papers on PRP have been published, the results are often contradictory. For HF-derived research [52,53,54], studies mainly include hair follicle stem cells (HFSCs) and dermal papilla cells (DPCs). Currently, treatment using HFSCs alone is progressing to clinical trials through preclinical models. However, the treatment with both HFSCs and DPCs is considered to be more reliable [15], which depends on DPCs as the signal center of the HF that plays an important role on HFSCs [53,55].

Previous studies showed that HF and keratinocyte components may promote hair regeneration when the amount of DPCs reaches a certain threshold [56,57]. Therefore, the enhancement of DPCs’ proliferation ability is an effective means to promote hair growth. Currently, three main types of strategies are available to promote hair growth through DPCs: Firstly, the proliferation of DPCs was promoted by medication. Kang et al. [58] found that mackerel-derived fermented fish oil (FFO) promoted DPCs’ proliferation via activating Wnt/β-catenin signaling for hair growth. Secondly, the proliferation of DPCs was promoted by overexpression or inhibition of endogenous genes. Our study falls into this category. Similar to our study, Wu et al. [17] found that Wnt10b promotes DPCs’ proliferation in Rex rabbits. In contrast, Yu et al. [59] demonstrated that mitotic arrest-deficient protein 2B (MAD2B) plays a negative role in T cell factor 4 (TCF4)-induced DPCs’ proliferation in humans. Thirdly, hair growth is promoted by increasing DPCs’ secretion of extracellular vesicles (EVs). Kwack et al. [60] reported that EVs derived from DPCs promote hair growth and hair regeneration by regulating the activity of follicular dermal and epidermal cells. Yan et al. [61] demonstrated that EVs’ microRNAs derived from DPCs mediate HFSCs’ proliferation and differentiation.

Despite the significant advances in the strategies, the molecular basis of promoting hair regrowth through DPCs still needs to be better understood. Hair regrowth resembles wound healing in that it requires a highly coordinated interplay between cell proliferation, cell differentiation, and cell migration [62]. The transformation of HF telogen and anagen stages depends on the cross talk of DPCs and HFSCs to produce the necessary activators [9,14]. Previous studies showed that DPCs promote hair regrowth by secreting Wnt/β-catenin [63], Notch [64], bone morphogenetic protein pathways (BMP) [65], and sonic hedgehog (Shh) [66] signaling molecules that communicate with epithelial cells [14]. In particular, the canonical Wnt/β-catenin signaling pathway plays a critical role in facilitating hair follicle entry into the anagen stage [67]. Similarly, during wound healing, studies showed that following cutaneous injury in mice, Shh levels rise, activating the hedgehog pathway and promoting hair follicle regeneration. Therefore, overexpression of Shh on the epidermis can lead to extensive HF regeneration in wounds, suggesting that activation of the Shh signal in Wnt-responsive cells can promote wound healing [68]. Based on these studies, although some studies confirmed that Tβ4 appears to affect the speed of hair growth via its effect on vascular endothelial growth factor (VEGF) expression in mice [30], we speculate that such growth-promoting effects of Tβ4 on DPCs might be associated with the Wnt/β-catenin signaling pathway. However, this speculation still needs to be confirmed by further experiments.

## 4. Materials and Methods

### 4.1. Ethics Statement

All experiments abided by the National Research Council Guide for the Care and Use of Laboratory Animals. The use of all tissue of Albas cashmere goat blocks for this study was approved by the Institutional Animal Care and Use Committee of Inner Mongolia University (Approval number: SYXK 2014-0002, in January 2014, Hohhot, China). The collection of all tissue samples from cashmere goats were accomplished at the YiWei White Cashmere Goat Limited Liability Company of Inner Mongolia (Ordos, China).

### 4.2. RNA Sequencing Alignment and Transcriptomic Analysis

We reanalyzed RNA raw datasets of the skin and SHF-DPCs samples of SHF anagen and telogen stages of Albas cashmere goat in our laboratory (three skin samples of SHF telogen, three skin samples of SHF anagen, one SHF-DPCs sample of SHF telogen, and three SHF-DPCs samples of SHF anagen stages) with the current analysis method. Simultaneously, we resequenced some samples on a HiSeq X10 platform (Illumina, San Diego, CA, USA) and the reads of the samples were generated. Then, all the raw data of each sample were analyzed (the reads with >5 base pair (bp) of adaptor sequences, >5% uncertain bases, or > 15% low quality bases (Q-score ≤ 19) were removed) to obtain the clean data (>6 Gb of each sample). The clean data were mapped back onto the Capra_hircus_ARS1 reference genome (ftp://ftp.ncbi.nlm.nih.gov/genomes/all/GCF/001/704/415/GCF_001704415.1_ARS1/GCF_001704415.1_ARS1_genomic.fna.gz, accessed on August 12, 2016) from the National Center of Biotechnology Information (NCBI) database using Bowtie2 v2.2.3 [69] and the mapping rate was 88.89–98.13%. The read count for each gene was calculated to assess gene expression using expected fragments per kilobase of transcript sequence per million base pairs sequenced (FPKM) value. DEGs of the samples were identified by DESeq2 v1.6.3 (q ≤ 0.05 and |log2_ratio| ≥ 1) [70].

### 4.3. Function Enrichment Analyses

We conducted GO enrichment and KEGG pathway analyses using the R package “clusterprofiler” [71]. GO terms or KEGG pathways with adjusted *p* < 0.05 were considered statistically significant and the biological process GO terms also visualized by “ggplot” (R package). The biological process GO terms also visualized by “GOplot” (R package) [72].

### 4.4. PPI Network Construction and Analysis of Selected DEGs

The STRING (functional protein association networks) database (http://string-db.org) (version 10.0) was used to construct the interactive relationships of the overlapping DEGs, and only the interactions with a combined score of >0.150 as the cut-off criteria were considered statistically significant. Subsequently, the PPI network was visualized using Cytoscape (version 3.7.1, National Institute of General Medical Sciences, Bethesda, MD, USA).

### 4.5. Gene Set Enrichment Analysis (GSEA)

We utilized the GSEA (http://software.broadinstitute.org/gsea/downloads.jsp, version 4.0.1, Broad Institute of MIT and Harvard, Cambridge, MA, USA) to perform the analysis for candidate genes. The gene sets used for GSEA were c2 KEGG gene sets (c2.cp.kegg. v6.2.symbols.gmt) from the Molecular Signatures Database. The threshold for the number of permutations was 1000. The criteria for statistically significant gene sets were nominal *p* < 0.05 and error discovery rate (FDR) <0.25.

### 4.6. Quantitative Real-Time PCR (qRT-PCR)

Total RNA of the samples was isolated using TRIzol reagent (RNAiso Plus*, TaKaRa Bio, Shiga, Japan) and complementary DNA (cDNA) was generated using PrimeScript RT reagent Kit with gDNA Eraser (TaKaRa Bio, Shiga, Japan) according to the manufacture’s protocol. The qPCR was conducted on a 7500 Real-Time PCR System (Applied Biosystems, Munich, Germany) with SYBR Premix Ex Taq II (TaKaRa Bio, Shiga, Japan). Relative gene expression was calculated using the comparative cycle threshold (2^−ΔΔCT^) method, with glyceraldehyde-3-phosphate dehydrogenase (GAPDH) as the endogenous control.

### 4.7. Vectors and Transfection

To overexpress Tβ4 (Tβ4-OE), the DNA sequences coding Tβ4 were cloned into separate eukaryotic expression vectors. To knockdown Tβ4 (Tβ4-KD), the short hairpin RNA (shRNA) targeting Tβ4 and the scramble shRNA were synthesized and ligated into the RNA interference (RNAi) vector. The Tβ4-OE and Tβ4-KD vectors were separately transfected into SHF-DPCs of Albas cashmere goat using Lipofectamine™ 2000 Transfection reagent according to the manufacturer’s protocol (Invitrogen, Carlsbad, CA, USA). SHF-DPCs were previously isolated and stored in liquid nitrogen.

### 4.8. Western Blot Analysis

Western blotting was performed with whole cell lysates in radioimmunoprecipitation assay (RIPA) lysis buffer mixed with SDS/PAGE sample buffer. The samples were separated by SDS-PAGE, and transferred to polyvinylidene fluoride (PVDF) membranes. Protein levels were analyzed via Western blotting using the indicated antibody. The images were visualized using the Tanon detection system (Tanon, Shanghai, China).

### 4.9. Cell Proliferation Assay

To assess proliferation, cells seeded in 96-well plates were determined by EdU using EdU Cell Proliferation Assay Kit (Ribobio, Guangzhou, China). According to the manufacture’s protocol, the cells were incubated with EdU for 12 h, then fixed in permeabilization buffer, washed with phosphate buffer saline (PBS), and stained with Apollo solution for 30 min. EdU-positive cells were observed using a Nikon AIR laser-scanning confocal microscope and the number of cells was calculated.

## 5. Conclusions

The proliferation of DPCs is an important source for the supplementation and growth of other hair follicle cells [61,73]. To determine whether Tβ4 affects the proliferation of SHF, we constructed Tβ4 overexpression and knockdown SHF-DPCs lines. The results showed that the overexpression of Tβ4 could effectively promote the proliferation ability of SHF-DPCs. Consistent with the above data, when the knockdown vector of Tβ4 was transferred into SHF-DPCs, the proliferation index of the cells decreased. In addition, we conducted a cell rescue experiment. When the SHF-DPCs knocked down Tβ4, they were re-transfected with Tβ4 overexpression vector and the proliferation ability of cells was rescued. These findings suggest that Tβ4 can promote the growth and development of SHF-DPCs and indicate that this molecule may be a valuable target for increasing cashmere production.

## Figures and Tables

**Table 1:** Summaries for five candidate genes.

Symbol	Full Name	Description
*Tβ4*	*Thymosin β 4*	Encodes an actin sequestering protein that plays a role in the regulation of actin polymerization. The protein is also involved in cell proliferation, migration, and differentiation.
*ARHGAP6*	*Rho GTPase activating protein 6*	Encodes a member of the Rho GTPase-activating proteins (rhoGAP) family of proteins that play a role in the regulation of actin polymerization at the plasma membrane during several cellular processes.
*ADAMTS15*	*ADAM metallopeptidase with thrombospondin type 1 motif, 15*	Encodes a member of the ADAMTS (ADAM metallopeptidase with thrombospondin type 1 motif) family. The encoded preproprotein is proteolytically processed to generate the mature enzyme. This gene may function as a tumor suppressor in colorectal and breast cancers.
*CHRD*	*Chordin*	Encodes a secreted protein that dorsalizes early vertebrate embryonic tissues by binding to ventralizing transforming growth factor-β- like (TGF-β-like) bone morphogenetic proteins and sequestering them in latent complexes.
*SPOCK1*	*SPARC (Osteonectin), cwcv- and kazal- like domains proteoglycan 1*	Encodes the protein core of a seminal plasma proteoglycan containing chondroitin- and heparan-sulfate chains. The protein’s function is unknown.

## Abbreviations

HF	Hair follicle
PHF	Primary hair follicle
SHF	Secondary hair follicle
DPCs	Dermal papilla cells
SHF-DPCs	Dermal papilla cells of secondary hair follicle
DEGs	Differentially expressed genes
